# Emerging Multi-omic Approaches to the Molecular Diagnosis of Mitochondrial Disease and Available Strategies for Treatment and Prevention

**DOI:** 10.2174/0113892029308327240612110334

**Published:** 2024-06-14

**Authors:** Faeze Khaghani, Mahboobeh Hemmati, Masoumeh Ebrahimi, Arash Salmaninejad

**Affiliations:** 1 Department of Pharmaceutical Biotechnology, School of Pharmacy, Guilan University of Medical Sciences, Rasht, Iran;; 2 Medical Genetic Research Center, Faculty of Medicine, Mashhad University of Medical Sciences, Mashhad, Iran;; 3 Department of Animal Biology, School of Natural Sciences, University of Tabriz, Tabriz, Iran;; 4 Regenerative Medicine, Organ Procurement and Transplantation Multi-Disciplinary Center, Razi Hospital, School of Medicine, Guilan University of Medical Sciences, Rasht, Iran

**Keywords:** Mitochondrial disease, omics, genomic, transcriptomics, proteomics, metabolomics

## Abstract

Mitochondria are semi-autonomous organelles present in several copies within most cells in the human body that are controlled by the precise collaboration of mitochondrial DNA (mtDNA) and nuclear DNA (nDNA) encoding mitochondrial proteins. They play important roles in numerous metabolic pathways, such as the synthesis of adenosine triphosphate (ATP), the predominant energy substrate of the cell generated through oxidative phosphorylation (OXPHOS), intracellular calcium homeostasis, metabolite biosynthesis, aging, cell cycles, and so forth. Previous studies revealed that dysfunction of these multi-functional organelles, which may arise due to mutations in either the nuclear or mitochondrial genome, leads to a diverse group of clinically and genetically heterogeneous disorders. These diseases include neurodegenerative and metabolic disorders as well as cardiac and skeletal myopathies in both adults and newborns. The plethora of phenotypes and defects displayed leads to challenges in the diagnosis and treatment of mitochondrial diseases. In this regard, the related literature proposed several diagnostic options, such as high throughput mitochondrial genomics and omics technologies, as well as numerous therapeutic options, such as pharmacological approaches, manipulating the mitochondrial genome, increasing the mitochondria content of the affected cells, and recently mitochondrial diseases transmission prevention. Therefore, the present article attempted to review the latest advances and challenges in diagnostic and therapeutic options for mitochondrial diseases.

## 1 INTRODUCTION

Mitochondria are the primary cellular organelles responsible for generating energy within the cell. They also play a crucial role in the regulation of cellular homeostasis, including processes related to cell death, oxidant generation, and calcium (Ca^2+^) balance [1]. The carbohydrates and fatty acids are efficiently transported into the mitochondria, where they are then oxidized to produce acetyl-CoA. This acetyl-CoA then undergoes further oxidation in the Krebs cycle (also known as the tricarboxylic acid cycle or TCA cycle) and the electron transport chain (ETC). This process of oxidative phosphorylation (OXPHOS) ultimately leads to the synthesis of ATP. In addition to being the primary energy producers, mitochondria are also the source of reactive oxygen species (ROS). These ROS play a role as signaling molecules and are required for maintaining cellular homeostasis [2].

Mitochondrial diseases are a group of clinically and genetically heterogeneous disorders caused by pathogenic variations in mitochondrial DNA (mtDNA) or nuclear DNA (nDNA) that encode mitochondrial proteins, resulting in dysfunction of mitochondrial ETCs and oxidative phosphorylation [3]. At least one in every 5000 live births and one in every 8000 adults is affected by mitochondrial disease, making it the most common inherited metabolic disorder [4].

A biochemical defect in OXPHOS enzymes causes a primary mitochondrial disease. More than 100 proteins, including 13 proteins encoded by the mtDNA and the rest encoded by the nuclear DNA, have a direct role in both biosynthesis and structural composition of OXPHOS and its components [5]. Human mitochondrial genomes are nearly exclusively inherited maternally. They are found in multiple copies within the cells, which may contain either exclusively identical copies of mtDNA (homoplasmy) or a mixture of mutated and unmutated mitochondrial genomes (heteroplasmy) [6]. Mitochondrial function is also affected by genes regulating mtDNA expression and replication. About 1500 genes have a role in healthy mitochondrial function, and up till now, pathogenic variants have been found in 309 mitochondrial metabolism genes. Additional pathogenic variants also likely still await discovery. The literature revealed that mitochondrial disease occurs *via* all existing patterns (recessive, X-linked, or dominant pattern), and it may be transmitted across cells and organs of the same person as well as through the germ lines of both parents, although a paternal contribution to the mtDNA pool appears much less frequent [7].

Dysfunctional mitochondria do not generate sufficient ATP to meet the energy demands of several organs, particularly tissues with high ATP consumption, such as skeletal and cardiac muscles, the liver, kidneys, the nervous system, and the endocrine system; moreover, a variety of cellular perturbations are more severely affected by ATP deficiency [8]. For example, in Leigh syndrome, deprivation of ATP due to OXPHOS dysfunction results in a wide range of manifestations [9], including failure to thrive, neurodevelopmental delay, ataxia, seizures, oculomotor apraxia, dystonia, apnea, and cardiomyopathy [10]. In mitochondrial diseases, energy deficiency associated with multiorgan dysfunction can manifest as cardiac and skeletal myopathies, hepatopathies, cognitive impairment, epilepsy, nephropathies, and endocrinopathies [8].

During the last decade, improvements in molecular and biochemical methodologies have enhanced our understanding of the etiology and the mechanism underlying mitochondrial disorders. However, progress in the development of therapies targeting these conditions has been lagging. As a result, most mitochondrial diseases are treated symptomatically without significantly altering their course [11].

For most mitochondrial disease patients over the last decade, dietary supplements and exercise – unless the patients are exercise intolerant - have been the only treatment options. CoenzymeQ10 (CoQ10) is the most effective supplement for primary and secondary forms of CoQ10 deficiency in only a few diseases [12]. On the one hand, individual types of mitochondrial diseases are comparatively rare, clinically diverse, pathogenically complex, etiologically heterogeneous, and sometimes still insufficiently studied [13]. On the other hand, understanding of mitochondrial diseases, their molecular genetic causes, and their pathomechanisms has progressed remarkably [14]. Thus, new treatment options are on the way as a result of these advances and emerging clinical trials.

In patients with suspected mitochondrial diseases, the extensive heterogeneity of the mitochondrial genome makes a certain genetic diagnosis and a pathogenic understanding difficult. In addition, clinicians may face more difficulties managing patients when multiple affected organs are involved [15]. In most cases, conventional candidate gene sequencing is preferred when the phenotype can be clearly defined. Still, the heterogeneity associated with a mitochondrial disease makes it worth using an unbiased approach early in the diagnostic process. As a result of high-throughput next-generation sequencing (NGS), whole genome sequencing (WGS) and whole exome sequencing (WES) have rapidly developed and become more affordable [16]. In consequence, the importance of discovering variants has shifted to an interpretation of function and clinical effects.

*MRPS16*, which encodes a subunit of mitoribosome, was the first gene associated with a mitochondrial disorder before NGS became widely available [17]. *MRPS22* was also identified before NGS, but other pathogenic gene variants, including mutations in *MRPL3, MRPL12, MRPL44, MRPS2, MRPS7, MRPS14*, *etc*. were later discovered using NGS [18].

In addition to improving diagnostic yield, WES demonstrated genetic variability in diseases, made it easier to identify monogenic mitochondrial mimics, and contributed to the advancement of understanding of mitochondrial biology, which has opened up new therapeutic possibilities [16, 17]. The potential of WGS lies in unbiased, simultaneous bigenomic sequencing, which incorporates non-coding regions and produces excellent mtDNA coverage depths [19].

Ever since the mitochondrial role in energy metabolism was first described in the 1950s and causal mtDNA variants were discovered nearly half a century ago, understanding of the diverse molecular causes and deleterious effects of pathogenic variants of the primary mitochondrial disease (PMD) has dramatically expanded. PMD is one of the most common types of metabolic disorders in newborns and arises due to mutations in the mitochondrial DNA of the ovum in an overwhelming number of cases or nuclear DNA of both the ovum and sperm [20]. With the goal of assisting healthcare professionals in the management of patients suffering from mitochondrial illness, a number of consensus recommendations and guidelines have been developed by clinical specialists from the United Kingdom (UK) and the USA [21]. In addition, the underlying gene defects in individuals are becoming more understood, which has increased the potential for developing specific therapeutic strategies. Recently, Australia and the UK have put specific legal regulations in place regarding mitochondrial replacement techniques (MRTs). In both of these countries, clinical trials on these techniques are currently allowed for cases where there is a high risk of the offspring developing a severe mitochondrial disease [22]. The present article attempts to review emerging genomic approaches, the latest advances, and challenges in molecular diagnostic and therapeutic options for mitochondrial disease.

## 2 THE IMPACT AND CHALLENGES OF HIGH THROUGHPUT STRATEGIES IN THE DIAGNOSIS OF MITOCHONDRIAL DISEASES

### 2.1 Genomics in Mitochondrial Disorders

Due to the progressive nature and lack of treatment options in mitochondrial diseases, techniques that prevent their transmission to the next generation and increase reproductive capacity are required. Since mitochondrial diseases are heterogeneous, they have genotypic and phenotypic variability and can overlap with other genetic conditions. Therefore, they are an ideal candidate group for non-targeted genetic diagnosis [23].

Since clinical diagnostic mtDNA testing is limited to examining a dozen common point mutations in well-known mitochondrial disorders, progress in this area in recent years was made by sequencing the entire mitochondrial genome. This approach has resulted in a significant improvement in diagnosing inherited mitochondrial diseases. Studies discovered that mutations in nuclear genes cause mitochondrial disease in three-quarters of children and one-third of adults [15]. As for other diseases, diagnostic options for the detection of nuclear mutations that lead to mitochondrial abnormalities, such as prenatal and pre-implantation genetic diagnostic tests, are available [24]. While the diagnostic challenges are greater for homoplasmic mutations, in which all copies of the mtDNA are mutated, most mutations are heteroplasmic (a combination of mutated and wild-type mtDNA) [25].

In addition, a defined set of candidate genes plays an essential role in mitochondrial function. Panels are designed for genes that are either known to cause mitochondrial disease or are predicted to play a crucial role in mitochondrial functions. These panels vary in diagnostic rate (7-31 percentage) and size, ranging from small panels that target 100 genes that cause complex I deficiency to more comprehensive panels that target both mt-DNA and over 1,000 nuclear genes that encode mitochondrial proteins [26]. However, there is a major drawback to the panels due to the continuous discovery of novel disease genes. Furthermore, since the panels cannot detect variations beyond the targeted genes, their accurate performance depends on correct clinical suspicion beforehand. In cases where the symptoms mimic another condition, the true diagnosis may not be detected [27].

Dual genomic sequencing enables accurate diagnosis in pediatric patients with mitochondrial diseases [28]. For instance, Leigh syndrome, which has a typical phenotype, can be caused by pathogenic variants within mtDNA or nDNA that are difficult to differentiate based on clinical features. With cost-effective, high-throughput sequencing technologies available, bigenomic sequencing, including whole exome and mtDNA sequencing, can be performed as an initial step for diagnosing patients with clinical manifestations of mitochondrial disorders [28].

Mitochondrial abnormalities are considered among the most difficult metabolic disorders to treat in children. NGS is a more recent technology to find new disease-causing genes and improve the clinical identification of rare inherited diseases. This technique has allowed great strides in understanding the molecular mechanism of these disorders of highly variable etiologies [29]. NGS includes WGS, WES, panel sequencing, and targeted mt-DNA sequencing. NGS facilitates a better understanding of genotype-phenotype relationships in mitochondrial diseases by enabling us to identify patients presenting atypically for their molecular diagnosis [30]. In addition, 85% of known monogenic disease-causing mutations are found in WES, addressing only the protein-encoding regions of the genome. NGS is performed in mitochondrial diseases with two approaches: targeted gene panels to sequence the mitochondrial exome and WES [5].

Total mt-DNA sequencing allows the heteroplasmy level to be monitored. For the study to begin, samples can be taken from peripheral blood and urine since these samples are readily available. However, heteroplasmy, proliferation, and the number of copies per tissue can vary based on the environment [31]. Therefore, a negative result does not indicate the absence of mutations, and tissue sampling should be performed. For adults with a higher probability of an mtDNA mutation diagnosis (75%), sequencing the mt-DNA from muscle biopsies usually precedes WES or WGS. In children, mt-DNA variants often appear in the urine and blood, but for confirmation, other tissue samples should be analyzed [27].

WGS and WES use short-read sequencing to align genome fragments to the human genome reference sequence. Many individuals with mitochondrial diseases can be genetically diagnosed using short-read exome sequencing. Still, the remaining individuals have an unknown cause, such as genomic rearrangements or copy-number variants (CNVs), splicing variants in deep intronic regions, variants in an unknown gene, or non-coding variants. Regardless of the underlying cause, further tests and techniques need to be applied to make a diagnosis. Clearly, WGS has been proven to be reliable for identifying CNVs and providing comprehensive exome coverage [32]. Recognizing strong genotype-phenotype correlations can often facilitate the diagnosis of similar cases. In pediatric mitochondrial diseases, for example, the discovery of recurrent duplications and deletions in *ATAD3* genes in cases of neonatal death led to the identification of pathogenic deletions, duplications, and missense variants of *ATAD3* in patients with mitochondrial diseases [33].

Certain genetic variants are not amenable to detection by WES, such as mosaic variants, genomic alterations including large and small deletions, small and large insertions, structural changes, repeat expansions, and deep intronic and regulatory variants. In fact, the undetectable deletions and insertions that are smaller or greater than 50 bp are important in the evaluation of mitochondrial diseases. WGS is mostly capable of distinguishing these changes with long-read WGS detecting deletions and insertions [27].

WES or WGS are two approaches to genetic diagnoses in an unbiased manner. When applying these approaches to mitochondrial disease, *a priori* candidate gene and the clinical heterogeneity of these disorders should be considered. However, the diagnostic yields of these studies rarely exceed 60%. While these genomic strategies cast a wide net, data analysis has historically been extensive. On the other hand, targeted panel NGS had the advantage of providing rapid diagnosis, with many diagnostic laboratories performing the technique locally [34]. With improved laboratory workflows and the streamlined and unified bioinformatic processes, clinical exomes and unbiased WES or WGS have reduced turnaround times, making them a powerful option for cases requiring rapid diagnosis. In addition, the advent of rapid genome sequencing (rGS) studies has improved turnaround times. A whole rGS diagnosis can be made in just 19.5 hours after receiving the sample. Rapid exome or genome sequencing offers unique advantages for severely ill newborns since it needs only a blood sample and eliminates the need for muscle biopsy and anesthesia [35].

Despite the recent popularity of WGS and WES using blood DNA as the primary method for mitochondrial disease diagnoses [36], many cases of causative variants remain unidentified either because of technological limitations (*e.g*., intronic variants in exome sequencing) [37] or lack of knowledge of the implicated pathology (*e.g*., genetic defects not yet associated with human disease) [38]. The mtDNA molecule exists in a circular form naturally. This circular structure can present challenges when using NGS techniques. This is because alignment tools are optimized to work with linear genomes and can struggle to properly map sequence reads that span the start and end of the circular mtDNA molecule. As a result, sequence reads in this area may get discarded by the alignment software. This can lead to diminished coverage of the control region. The control region's repetitive and highly variable DNA sequence further compounds the challenge, as these features can also contribute to the suboptimal alignment of reads in this area. Furthermore, the control region is typically the location where primers are placed for mtDNA enrichment during PCR amplification. This means the control region is often underrepresented in the sequence reads obtained from PCR-enriched samples [39].

A WGS approach within the diagnostic pathway combined with CNV analysis tools, such as Exome Depth [40], is one way to address this possibility. Long-read sequencing technologies, such as those developed by Oxford Nanopore Technologies (ONT) and Pacific Biosciences (PacBio), provide an efficient means for detecting large-scale genetic variations [41]. A de novo *CLN6* deletion was detected in monozygotic twins who presented with myoclonic epilepsy, making these tests successful in diagnosing some patients [42]. Until now, there has been no objective measurement of mitochondrial disease likelihood. Present scores, if calculated rigorously, may assist in identifying high-risk patients. Although this variation exists, the diagnostic rate of WES varies from 24 to 68% for rare disorders. Mitochondrial diseases, being rare disorders, can be diagnosed very well using WES, making it a well-suited first diagnostic choice. [43].

### 2.2 Mitochondrial Transcriptomics

RNA sequencing (RNA-seq) provides data about transcript abundance in a specific tissue [34] that can help with prioritizing variants in WES and WGS by eluding consequences of variants of uncertain significance (VUSs) [5]. It provides information about the effects of VUSs of any type, especially synonymous or non-coding [5], located within introns and regulatory regions [34].

Earlier studies have used RNA-seq to detect the pathogenic consequence of some intronic and exonic variants because it provides valuable data for prioritizing VUSs. There are some examples of mitochondrial disorders for which the cDNA sequence was required to reveal the pathogenic mutation. For instance, a mutation in *TAFAZZIN*, which is a gene encoding taz protein associated with x-linked Barth syndrome [44, 45], was first predicted to be synonymous (NM_000116.3: p.Gly116Gly) [3, 4] and was therefore excluded from the WES analysis [3, 4]. Then, further investigations showed that this variant activates a cryptic splicing donor site and results in an altered mRNA sequence, resulting in the deletion of 8 amino acids [3, 4]. The other example is a mutation that was first detected by WES and predicted to be missense, NM_024120.4:c.327G>C in *NDUFAF5* gene (p.Lys109Asn), which encodes a mitochondrial protein involved in complex I assembly (mitochondrial arginine-hydroxylase NDUFAF5) [37]. However, further investigations demonstrated the role of NM_024120.4:c.327G>C in interfering with splicing and exon skipping [37]. In addition to exonic variants, the pathogenic effect of some intronic variants identified by WGS was explained using RNA-seq data. For example, chr2:g.240964793_240964817del is an intronic variant that was detected in two patients with isolated complex I defect in skeletal muscle and a decrease in expression of mRNA transcripts [46]. WGS detected this 5’ UTR deletion variant within the *NDUFA10* gene, which encodes a mitochondrial complex I subunit (Complex I-42kD) [46]. Hence, this method contributes to prioritizing intronic or exonic VUSs, which affect either the expression or the splicing pattern.

There are three strategies to prioritize disease-causing variants and identify genetic defects of a condition [5]. Prioritizing disease-causing variants is conducted by analyzing improper expression, monoallelic expression (MAE), and improper splicing [46]. Expression outliers are genes with expression levels outside of the normal range. The reasons for this aberrant expression are rare variants in regulatory enhancer regions and promoters [47], in addition to post-transcriptional mechanisms [5]. The second strategy, MAE (when one allele is silenced), can be identified by RNA seq [5]. MAE of rare heterozygotic variants contributes to prioritizing a variant in a condition that is usually inherited by an autosomal recessive pattern [46]. Aberrant splicing, the last strategy, might be a consequence of variants that are located close to splice sites [5]. They might affect exons and result in exon creation, skipping, truncation, and extension as well as intronic inclusion [46, 48]. These variants affecting splicing are the most recurring abnormal findings in RNA-seq [46]. By taking advantage of gene splicing and expression patterns obtained from RNA-seq data, pathogenic variants can be prioritized.

The number of detectable transcripts might be variable in different tissues. Hence, RNA-seq evaluates transcript abundance in a specific tissue. The analysis of 338 mitochondrial genes in 3 different tissues demonstrated that 60% of genes are detectable in whole blood with an FPKM>1 (fragments per kilobase of transcript per million mapped fragments), while in cultured fibroblasts and muscles, more genes are detectable (90% and 86%, respectively) [27]. Considering disease genes in the Online Mendelian Inheritance in Man (OMIM) database, around 45% of genes in whole blood and approximately 75% in fibroblasts and muscles are expressed with FRKM >1 [49]. Therefore, selecting the target tissue as the sample is an important step in RNA sequencing.

In addition to transcript abundance, the RNA-seq diagnostic rate varies according to the sample tissue (8%-36%) [46, 48]. In fact, the RNA-seq diagnostic rate depends on both the disease and the sample. RNA sequencing resulted in a diagnosis of 15% of patients with mitochondrial diseases who had a negative WES result [46]. Only a portion (10%) of RNA-seq outliers is reproducible in other tissues [50], although monoallelic expression outliers are persistently detectable [50, 51]. Given that, tissue selection is an important step [34]. Considering variable splicing in tissues, tissue-specific splicing should be evaluated before tissue selection [34]. Besides, some sources, such as the expression atlas [52], PAGE [53], and GTEx [51], can be used for tissue selection. Therefore, to obtain the best result, it is crucial to select a specific tissue considering its expression and specific splicing pattern [54, 55].

### 2.3 Mitochondrial Proteomics

Proteomics is the analysis of a large number of proteins for further purposes, including evaluating a gene’s presence and function [56], supporting the data obtained through other omics such as transcriptomics [57], and studying posttranslational changes [56].

The ability of proteomics to detect protein destabilization as an abnormally reduced abundance of the protein compared to a reference sample [58] makes it suitable for the analysis of mitochondrial disorders. The most common mutation occurring in mitochondrial diseases is missense, which might not affect protein function but might destabilize protein [27] or disrupt its interaction with other proteins [27]. In contrast to transcriptomics, proteomics gives direct information on proteins [57] and can present advantages in comparison to transcriptomics [57]. For example, it enables an analysis of the consequence of missense variants, the most frequent type of pathogenic variants found in patients with mitochondrial disorders [34]. Almost 40% of missense mutations lead to protein destabilization [59]. Therefore, proteomics can contribute to the diagnosis of unsolved cases.

Besides, the ability to assess a vast number of proteins rapidly makes proteomics more beneficial in comparison to more traditional, time-consuming methods such as western blotting [34], which is also dependent on the availability of specific antibodies. Quantitative proteomic analysis can be helpful in detecting the origin of defects in cellular pathways in patient samples [60]. Proteomic studies have identified two novel genes related to mitochondrial diseases: *MRPS34* and *PTCD3* [60, 61]. In fact, the VUS in the *MRPS34* gene, encoding a small mitoribosomal subunit protein (MRP-S34), leads to the destabilization of the gene product and the following defect in mitochondrial translation that was detected as a remarkably reduced level of all small mitoribosomal subunits [60]. Likewise, quantitative proteomic analysis of fibroblasts of patients with a significant reduction in mitochondrial complex I and IV levels and activities, as well as mitochondrial translation defects, revealed that variants in *PTCD3* (*MRPS39*) [61] encoding a mammalian mitochondrial ribosomal supernumerary protein (MRP-S39) [62] resulted in a decrease of small mitoribosomal subunits [61]. Another example is the study by Kremer *et al*., where the level of respiratory chain complex I assembly factor TIMMDC1 (protein M5-14) [46, 63] is reduced or ablated in the proteome due to a decrease in its RNA expression in cases with TIMMDC1 VUS [46]. Kremer *et al*. not only confirmed the significant role of TIMMDC1 in respiratory chain complex I assembly but also demonstrated that its deficiency is disease-causing [46].

Although proteomics can contribute to revealing the consequence of some missense mutations, it is not applicable for most missense variants that do not result in protein degradation [27]. Since these mutations encompass approximately 60% of missense variants, which do not affect protein stability or folding [59], proteomics approaches are limited in their usefulness for the detection of disease-causing variants [27], and other techniques are required in many cases.

### 2.4 Mitochondrial Metabolomics

Some metabolites, such as alanine, pyruvate, creatine kinase, lactate, acylcarnitines, thymidine, deoxyuridine, and organic acids, are measured in urine and blood samples as classical biomarkers of mitochondrial disorders [27, 64]. Although biochemical tests often have limited sensitivity and specificity for diagnosis [27], being a noninvasive approach makes them potentially appropriate for “patient monitoring”, which is an important challenge regarding mitochondrial disorders and indicates checking progression besides response to therapy [65]. Thus, it is considered as a research priority [66] and is being investigated firmly using high-throughput tools like metabolomics [67].

Metabolomics quantifies more than thousands of metabolites *via* mass spectrometry (MS) in specific tissues [59]. Besides monitoring the patients, this technique contributes to the diagnosis of patients with certain mitochondrial disorders, newborn screening [68], and inborn disorders discrimination. Likewise, its high value in diagnostic procedures of some genetic disorders, such as inborn errors of metabolism (IEM), has been proven [69].

Numerous conventional biomarkers can be applied to support the diagnosis of mitochondrial diseases. The metabolic fingerprints of OXPHOS deficiency are considered the most useful biomarkers in the diagnosis of mitochondrial disorders. For example, the level of fibroblast growth factor 21 (FGF21) is a possible biomarker for mitochondrial-based myopathy [64]. Methylmalonic aciduria and 3-methylglutaconic aciduria are two mitochondrial disorders that can be identified in children by measuring levels of organic acids in urine [70, 71]. Gaining a comprehensive understanding of the biomarkers associated with mitochondrial diseases is essential for making progress in earlier diagnosis of these conditions, enhancing the management of patients, and facilitating the development of targeted therapies [72].

Metabolomic assessment can be conducted through several methods using different samples. It can be performed individually or alongside other tests to result in the diagnosis of a condition. For instance, metabolomic analysis *via* MS-based methods is utilized to perform newborn screening for two fatty acid oxidation disorders that are treatable [73, 74]: very long-chain acyl-coenzyme A dehydrogenase deficiency (VLCADD) and medium-chain acyl-coenzyme A dehydrogenase deficiency (MCADD) [75, 76]. Moreover, a plasma sample analysis by liquid chromatography quantitative time-of-flight (LC-QTOF) mass spectrometry has been performed to differentiate between a variety of inborn metabolism disorders [69]. Furthermore, a combination of genetics and metabolomics can aid in diagnosing patients with complex mitochondrial conditions. Since some metabolites are specific [77], detecting them by gas chromatography-mass spectrometry (GC/MS) urinary acid analysis can indicate defects in specific metabolic pathways, and some genes can be suggested for further analysis [34]. For example, elevated methacrylyl-CoA and acryloyl-CoA-related metabolites in patients with short-chain enoyl-CoA hydratase (SCEH) deficiency suggested that *ECHS1* gene encodes the mECH1 protein analysis [78]. Although no potential pathogenic variant was detected *via* WES, sanger sequencing identified a pathogenic variant NM_004092.3:c.538A>G, p.(Thr180Ala) in a low-depth read region. This case also indicates the importance of validating the results of NGS by Sanger sequencing [34].

In addition to contributing to diagnosis, metabolomic findings combined with transcriptomic insights can support genomic data in order to classify a novel variant. For instance, metabolomic findings including C3-carnitine and C3-/C2-carnitine ratio in the blood samples and increased urinary amino acids indicated elevated excretion of methyl citrate and 3-hydroxy-propionate [79] that aided in verifying a novel missense mutation, NM_000282.2:c.1427G>C p.(Arg476Pro), within *PCCA* gene, which codes PCCase subunit alpha protein [79]. According to these metabolic findings, genetic results, and *in silico* protein modeling, the mutation was classified as “likely pathogenic”, which corroborated the diagnosis [79].

**Fig. (1):**
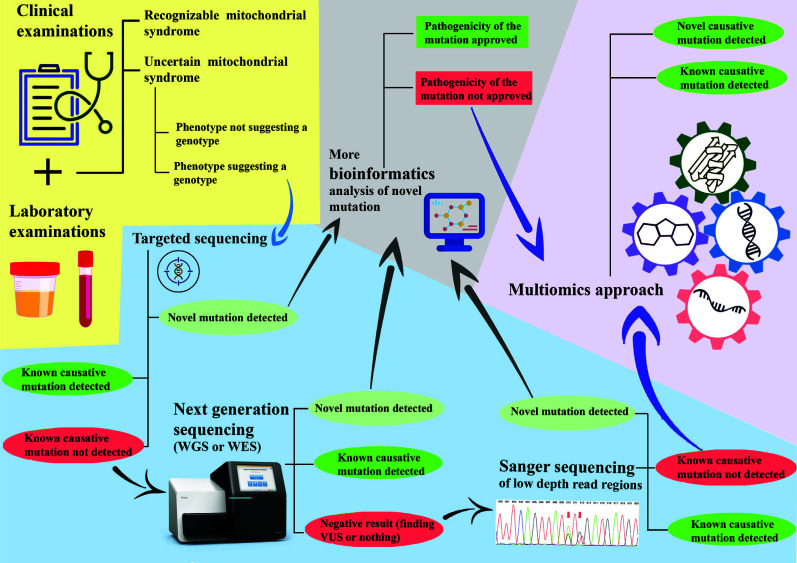
A comprehensive proposed diagnostic approach for mitochondrial diseases.

It is obvious that the multi-omic approach, a combination of omic approaches such as WES or WGS, transcriptomics, and proteomics referenced to each other, can potentially enhance the diagnostic rate and result in the identification of new genes related to mitochondrial diseases (Fig. **1**) [17]. Therefore, such a strategy contributes to an understanding of the pathogenesis of the disease [17]. Recently, Aboulmaouahib *et al.* analyzed the inverted mitochondrial genome-wide association between concentration of metabolites and mitochondrial nucleotide variants (mtSNVs). To find the genetic variations associated with metabolite profiles, they employed the entire sequenced mitochondrial genome and 151 metabolites from 2718 people. As a result, the strongest association was found for mt715G > A, which was located in the MT-12SrRNA having a metabolite ratio of C2/C10:1, which was associated with the metabolite ratio acetylcarnitine/decenoylcarnitine (*P*-value = 6.82^*^ 10 ^−09^, β = 0.909) [80]. Results showed that an alteration of C2/C10:1 serum level enhances the ratio of heteroplasmy at mt715G > A, which, in turn, can result in MT-12SrRNA dysfunction and might even affect insulin regulation. C10:1 is measured to be a fatty ester lipid molecule. This metabolite was discovered to be related to type 2 diabetes and prediabetic states [81].

## 3 THERAPEUTIC OPTIONS OF MITOCHONDRIAL DISEASE

### 3.1 Small-molecule Therapies (Pharmacological Approaches)

One of the common existing therapeutic approaches for patients with mitochondrial disorders is consuming dietary supplements, including vitamins and cofactors [82], which have different roles in the treatment of these disorders. For instance, some supplements, such as vitamin B1, serve as a cofactor, while others might be a mitochondrial substrate (L-carnitine), functioning as an antioxidant and/or increasing respiratory chain flux [82]. Antioxidants are often used for treating patients with mitochondrial diseases because, in most mitochondrial disorders, pathogenic cellular damage might occur as a result of respiratory chain dysfunction following the production of extra ROS [82].

Coenzyme Q10 (CoQ10), or ubiquinone, is a key component of the ETC that transfers electrons from complexes I and II to complex III [83] and functions as an endogenous antioxidant that increases respiratory chain flux. CoQ10 increases electron transfer and improves clinical outcomes resulting from CoQ10 deficiency [84], which occurred due to defects in enzymes responsible for CoQ10 synthesis [85]. It is suggested to enhance electron flow through the ETC in other mitochondrial diseases [83]. Many clinical trials have investigated the efficacy of CoQ10 and its analogs (idebenone and vatiquinone) in the treatment of several mitochondrial disorders [86], with idebenone (analog of CoQ10) displaying a higher efficacy. The compound has been utilized in treating Leber hereditary optic neuropathy (LHON), and findings suggested that it can prevent the progress of vision loss [87]. In addition, a follow-up study showed that drug effects are partially permanent and persist even after treatment [87]. The effectiveness of idebenone in protection from loss of vision was further demonstrated in an independent study [88].

Vitamins C and E, as well as their analogs, are other antioxidants that might be efficient in mitochondrial disease treatment. Supplementation with Trolox (ornithylamide hydrochloride), which is a vitamin E analog, resulted in ROS reduction and an increase in mitochondrial complexes I and IV, and citrate synthase activity in fibroblasts derived from patients with Leigh syndrome [89]. However, the efficacy of antioxidants remains argumentative [82] as there is controversial evidence on the efficacy of antioxidants. A review of treatment strategies for mitochondrial disorders showed little evidence supportive of the beneficial effects of vitamins or cofactor supplementation [90]. Therefore, the data concerning the usefulness of these compounds is inconclusive.

An antioxidant that targets the redox systems of glutathione (GSH) or thioredoxin (Trx) is an agent with significant therapeutic potential [91]. Vatiquinone, also called vincerinone, EPI-743, or α-tocotrienol quinone, is a vitamin E analog that elevates GSH levels and upgrades GSH detoxification pathways [92]. Furthermore, RP103 (cysteamine bitartrate delayed-release) has the same function. The beneficiary of these two compounds is being tested [93].

Sonlicromanol (KH176) is a novel agent that affects the Trx system. It reduces ROS-induced cell death by interacting with peroxiredoxins (Prxs) and enhancing their peroxidase activity [49]. Currently, sonlicromanol is being investigated in three double-blinded, placebo-controlled (DBPC) studies in phase II clinical trials (KHENERGYC, KHENERGY, and KHENEREXT). The pharmacokinetics, efficacy, and safety of sonlicromanol were assessed in different populations: adults with mitochondrial disorders due to mitochondrial m.3243A>G in the KHENERGY study [94] and a separate group of children bearing genetically confirmed mitochondrial disease in the KHENERGYC study [95]. The results showed that sonlicromanol had a positive effect on depressive symptoms in the populations in the KHENERGY study [94]. Alpha-lipoic acid is a potential antioxidant [96] and is often used alongside other antioxidants [97-99] and commonly included in “mitochondrial cocktails” [100]. It also has an important role in pyruvate dehydrogenase and ketoglutarate dehydrogenase function, although it is uncertain if nutritional lipoic acid can be activated for attachment to these complexes in mitochondria [101].

Cysteine has a crucial role in glutathione (an intracellular antioxidant) synthesis. In fact, cysteine donors, including cysteamine bitartrate and N-acetylcysteine (NAC), can reinstitute glutathione levels; therefore, using them as supplements can eliminate more ROS in mitochondrial disorders with glutathione deficiency [83]. Recently, the investigation of a safe dose of N-acetylcysteine commenced in a group of patients with mitochondrial disorders in a phase I clinical trial (https://ClinicalTrials.gov/show/NCT05241262j). In Table **1**, some important current clinical trials in mitochondrial disorders are summarized.

Thiamine (vitamin B1) increases the activity of pyruvate dehydrogenase due to enhancing pyruvate oxidation and the availability of reduced cofactors [83]. Supplementation with this compound was shown to exert beneficial effects on lactic acidosis and myopathy in a family suffering from thiamine deficiency and MELAS syndrome [102]. Thiamine has been used in combination with vitamins C and E and CoQ10 carnitine in patients with adult-onset Leigh disease, demonstrating neurological abnormalities and resulting in significant improvements [103]. OMT-28, a synthetic compound that mimics 17,18- epoxyeicosatetraenoic acid (17,18-EEQ), is being studied as a potential treatment for mitochondrial dysfunction (Table **1**). This drug is related to the activity of Omega-3 fatty acids and is currently being studied in Phase 2 clinical trials (NCT05972954). OMT-28 has been shown to improve calcium handling and mitochondrial function in heart muscle cells (cardiomyocytes) and reduce the signaling of pro-inflammatory molecules [104].

### 3.2 Manipulating the Mitochondrial Content of Patient Cells

Most patients with mitochondrial disorders suffer from a lack of energy [82]. It has been discussed that increasing the mitochondrial content of patient cells may be beneficial and improve disease symptoms [105]. For example, mitochondrial biogenesis was suggested as a therapeutic strategy for LHON patients [106] since enhanced levels of mitochondrial mass affected incomplete penetrance in the patients [106]. Also, studies have shown that acipimox, bezafibrate, omaveloxolone, KL1333, and REN001 (PPARδ) have the potential to increase mitochondrial biogenesis [107, 108]. In addition, mitophagy is the other factor controlling the level of mitochondrial mass that can be reduced [109]. It is widely accepted that various forms of exercise can act as a potent stimulant for mitochondrial biogenesis [110], which is beneficial in some patients [111]. However, a recent observation in ND6, CO1, and ND5 mice demonstrated that the benefit of endurance exercise depends on mitochondrial variations [112], which may highlight the role of genetic analysis in deciding the most appropriate strategy for treatment. In addition to exercise, various small molecules were investigated for their effect on mitochondrial proliferation and mitophagy [109]. Some of these compounds upregulate mitochondrial biogenesis. An example is PPAR-gamma coactivator1-alpha (PGC1α), which is a member of the peroxisome proliferator-activated receptor family and the critical mediator of mitochondria proliferation [82]. This nuclear receptor operates as a transcription cofactor that begins expressions of other factors important for mitochondrial dynamics and homeostasis [113] by interacting with a variety of transcription factors such as respiratory factors (NRF1 and NRF2) and PPARs (PPAR α, β, and γ) [82], which enhance the expression of oxidative phosphorylation (OXPHOS) genes and fatty acid oxidation-related genes (FAO), respectively [114].

The only treatment approved by the FDA for patients with mitochondrial disorder (Friedreich’s ataxia) is Omaveloxolone (SKYCLARYS). This treatment functions by protecting Nrf2 and prevents its degradation by binding to a protein called Keap1 (Kelch-like ECH-associated protein 1) [115]. Omaveloxolone has been shown to improve the function of complex 1 in the electron transfer chain, thereby restoring mitochondrial function [115]. It is currently being used to treat patients with Friedreich’s ataxia. The FDA's approval of Omaveloxolone suggests that it may be more effective than other drugs currently in development for mitochondrial diseases. However, there are differing opinions on its clinical effects, and the strength of the supporting data is still a topic of debate [116].

The PGC1α activity is regulated by Sirtuin (Sirt1) and AMP-activated protein kinase (AMP) that increase PGC1α activity by deacetylation and phosphorylation, respectively [117]. Given these facts, Sirt1, AMP enzymes, and other agents involved in the biogenesis pathway can be modulated by drugs [82, 118]. The overexpression of key mediator of mitochondrial proliferation, PGC1α, has been shown to lead to an increase in COX activity in a mouse model of cytochrome c oxidase assembly deficiency [119]. Besides, an improvement in the skeletal muscle and heart function associated with PGC1α was reported in a mutator mouse model [120]. Despite the promising results of preclinical studies, the regulation of this transcription factor is complex due to many forms of posttranslational modification [121]. Therefore, PGC1α could not be an appropriate target for pharmaceutical intervention [109]. Bezafibrate is a panPPAR activator utilized for treating hyperlipidemia [109]. There is controversial data about its efficacy for mitochondrial disorders. In some studies, bezafibrate has shown promising results in fibroblast of patients with mitochondrial disorders and a Deletor mouse model [120, 122]. However, a different mouse model exhibiting COX deficiency did not demonstrate any evidence of induced mitochondrial proliferation [120, 122, 123].

Upstream agents involved in the mitochondrial proliferation pathways, such as AMPK and Sirt1, may be targets for modulation by drugs as well. The activator of AMPK, 5-aminoimidazole-4-carboxamide ribonucleotide (AICAR), is an analog of adenosine monophosphate that was used in some cytochrome C oxidase (COX) deficient mouse models to enhance activities of respiratory chain complex [119]. Among three mouse models (Surf−/−, Sco2KOKI, and ACTA-Cox15−/−), only the knockout/knockin Sco2 mouse (Sco2^KO/KI^) model showed improvement in motor performance [119]. AICAR can also stimulate Sirt1 and, therefore, can be a potential therapeutic option for mitochondrial disorders [119, 124, 125]. Metformin, an analog of biguanine that functions by preventing complex I [126-128] or mitochondrial glycerophosphate dehydrogenase [129], is considered a putative activator of AMPK [130], although its contribution in mitochondrial biogenesis is not clear yet. Another stimulator of mitochondrial biogenesis is resveratrol, a natural plant polyphenol that activates both AMPK and stress-induced phosphoprotein 1 (STIP1) [131]. Resveratrol was tested promisingly in human fibroblasts and animal models and reported as an activator of mitochondrial biogenesis [132]. However, in another study on human fibroblasts, it did not increase OXPHOS activities [133].

In conclusion, despite strong evidence supporting the potential role of mitochondrial proliferation in restoring mitochondrial function, no adequate data support small molecules as a therapeutic option for mitochondrial biogenesis [109]. Since exercise, the only well-documented existing approach to increase mitochondria, is only beneficial in some patients, more studies in this field are required to yield new therapeutics appropriate for more patients.

### 3.3 Inducing Mitochondrial Turnover (Activation of Mitochondrial Biogenesis)

In mitochondria, biogenesis is tightly regulated, depending on both nuclear and mitochondrial factors. For mitochondrial gene transcription and translation to occur synchronously, the mitochondrial and nuclear genomes must be transcribed and translated at the same time. A respiratory chain complex, for example, is formed both by nuclear and mitochondrial components. When the composition of these complexes is unbalanced, it results in proteotoxic stress, which activates mitochondrial turnover mechanisms [134]. In mitochondrial biogenesis, a cellular process takes place when the mitochondrial genome cooperates with the nuclear genome to form “new” mitochondria. The balance between mitochondrial biogenesis and mitophagy defines mitochondrial turnover [135].

In the molecular mechanisms involved in protein turnover as well as mitochondrial biogenesis, first, muscle mass is determined largely by protein turnover, which is a delicate balance between protein synthesis and protein degradation. The phosphatidylinositol 3-kinase (PI3K)-Akt pathway can be viewed as a key signaling pathway to enhance skeletal muscle mass by improving abnormal protein turnover where protein breakdown exceeds protein synthesis. As a result of activating the PI3K-Akt pathway, translation is enhanced through the phosphorylation of mTOR, which works by inhibiting the Forkhead Box O (FoxO) protein, which in turn suppresses the proteolysis-related system known as the ubiquitin-proteasome [136]. In addition, mitochondria are key organelles for generating chemical energy in muscles [137]. In addition to accelerating the proteasome-ubiquitin system, FoxO activates the autophagic-lysosomal system, which is responsible for breaking down misfolded proteins and organelles, especially mitochondria [138]. PGC-1 α, on the other hand, is a major target protein for improving mitochondria function because it induces mitochondrial biogenesis, drastically increasing mitochondrial quality and quantity [139]. There are three variants in the PGC-1 superfamily, all of which are directly included in regulating metabolic gene expressions, such as those that regulate mitochondrial proteins and fatty acid oxidation [140].

Under stress and aging, mitochondrial function is disrupted by damage accumulating within the organelles. Cells have complex systems to repair damage and save organelles as the first line of defense. The mitochondrial ATP-dependent proteases identify misfolded and/or oxidized polypeptides in different mitochondrial compartments and cause their proteolysis, while the cytosolic ubiquitin-proteasome system (UPS) targets outer mitochondrial membrane (OMM) proteins selectively [141]. Furthermore, activating the mitochondrial unfolded protein response (UPRmt) triggers a retrograde signal to the nucleus, which increases the expression of mitochondrial chaperones and ameliorates mitochondrial proteotoxic stress [142].

The formation of mitochondrial-derived vesicles (MDVs) is another interesting repair mechanism recently discovered. These MDVs are generated by the enclosing of mitochondrial components in mitochondrial membranes, followed by scission events. These MDVs are released into the cytosol and subsequently degraded by lysosomes [143].

In cells or animals with mitochondrial dysfunction, an increase in mitochondrial mass has often been suggested to beneficially affect the progress of disorders [144]. To achieve this, it is necessary to positively regulate mitochondrial biogenesis and reduce mitophagy. A number of nuclear receptors, such as the peroxisome proliferator-activated receptor (PPAR) family and transcriptional coactivators of one of these receptor isoforms (and many other nuclear receptors), are now recognized as main mediators of mitochondrial biogenesis that initiate expression of several important mitochondrial factors [145, 146]. In addition to treating many aspects of mitochondrial dysfunction, the drug rapamycin has shown promise as an anti-proliferative and anti-inflammatory drug. Rapamycin was first isolated from the soil bacterium Streptomyces hygroscopicus. In mitochondrial myopathy, its target is the mTORC1 protein, which is a part of the “mammalian target of rapamycin” (mTOR) complex and is linked to cellular homeostasis. Researchers have shown that rapamycin may affect autophagic flux and lysosome biogenesis through the inhibition of the mTORC1 pathway. Rapamycin reverts the progression of mitochondrial myopathy in mice [147]. Because of the defective COX assembly, OXPHOS can never be fully restored. The use of rapamycin as a treatment results in metabolic reprogramming, suggesting that other approaches that mimic this reprogramming process might be beneficial. Rapamycin treatment partially restores mitochondrial function in these models, and it has been used in many clinics for many years. Although it may help treat human mitochondrial disease, this medicine has substantial side effects other than immunosuppression that limit its use [148].

Studies have also shown that long-term exposure of cells to low concentrations of nitric oxide (NO) in a culture medium stimulates mitochondrial biogenesis [137]. In this process, cGMP is generated when “soluble” guanylate cyclase (sGC) is activated by NO, and the nuclear respiratory factor 1, mitochondrial transcription factor A (TFAM), and peroxisomal proliferation-activated receptor coactivator 1 (PGC-1) are also expressed at a higher level [137]. However, there are limited data that support the use of small molecules as therapeutics for mitochondrial biogenesis, even though there is a strong argument for increased mitochondrial biogenesis. Training and exercise are the most promising ways of increasing mitochondrial mass. They are safe across various systemic outcomes and do not appear to harm the body in any way [149].

### 3.4 Mitochondrial Augmentation Therapy and Mitochondrial Transplantation

A recent study reported a therapy for the treatment of patients with large-scale mtDNA deletion syndrome by supplying them with autologous CD34^+^ hematopoietic stem and progenitor cells (HSPCs) augmented with functional mitochondria derived from cells from the patients’ mothers [150]. In a compassionate use study, patient CD34^+^ HSPCs were augmented with mitochondria isolated from maternal peripheral blood mononuclear cells by *ex vivo* incubation. Results indicated improvement in the patient’s condition based on clinical and quality-of-life assessment parameters by caregivers. The number of patients in this study was small, and therefore these results should be interpreted with caution. However, several other recent reports indicated that isolated, active mitochondria are readily taken up into tissues even *in vivo* and can “home” into recipient cells where they can support cellular function in a process termed “mitochondrial transplantation” in humans and a porcine model [150-154]. In addition, another study evaluates the efficacy of the MNV-201, a type of cell therapy created using mitochondrial augmentation technology (MAT) (NCT06017869). These cells are made up of the individual's own CD34+ progenitor and hematopoietic stem cells (HSPCs) that have been enriched with mitochondria derived from a placenta donor.

### 3.5 Manipulating the Mitochondrial Genome

The copy number of the mitochondrial genome varies from none (erythrocytes) or very few (in early embryonic cells) to tens of thousands of copies per cell in oocytes, depending on the type of the organism [155].

The molecular replication of mtDNA by nuclear DNA-encoded polymerase γ is largely independent of the cell cycle [156]. As a result of double-strand break production, linear mtDNA molecules get degraded and lost. Therefore, to reduce mutant mtDNA levels, the use of mtDNA-mutation-specific nucleases specifically targeted to mitochondria has been suggested as a therapy approach to reduce mutant mtDNA in patients harboring heteroplasmic genomes. Because mtDNA levels are typically controlled by cells, this strategy is especially effective. MtDNA is replicated if the number of copies of mtDNA suddenly decreases.

A mitochondria-targeted restriction endonuclease (mitoRE) has proven to be an effective tool for manipulating mtDNA heteroplasmy. However, its application is limited to the generation of a unique restriction endonuclease site by the mutation of interest. ZFNs and TALENs, which are programmable gene editing tools, were created to overcome this limitation [157-159]. For mitoTALENs and mtZFNs to be used in clinical practice, gene therapy tools must be used. Viruses have evolved to target specific cell types without being detected by the host's immune system. Gene delivery with adenoviral vectors can be efficient in dividing and non-dividing cells but can result in insertional mutations. There is often a transient and primarily liver-based limitation on gene expression caused by the immune elimination of infected cells *in vivo* [160]. Although the Herpes simplex virus is capable of delivering large amounts of exogenous DNA, some significant challenges remain including insertional mutagenesis, cytotoxicity, and maintaining transgene expression [161]. Mitotic and post-mitotic cells can be infected by adeno-associated viruses (AAV), but their genetic cargo can only be delivered to a limited degree. AAVs have a limited immunological profile and cannot integrate into the host nucleus [162]. However, gene therapy by recombinant AAV vector ND4 (rAAV2) was found to be safe and well tolerated in a phase I clinical trial of nine individuals with LHON [163]. The Wuhan group initiated the first clinical trial (phase 1/2/3) of NR082, AAV2-ND4 in China in 2021 (NCT04912843). Part 1 of the study involves dose escalation, while Part 2 is a randomized, double-blind, controlled study on patients aged over 12 and under 75. The purpose of the study is to confirm the effectiveness and safety of NR082. The NFS-02, rAAV2-ND1 vector, is also composed of a recombinant AAV2 vector that delivers the human wild-type mitochondrial ND1 gene. This drug is administered *via* the intravitreal route to treat LHON caused by the G3460A mutation within the ND1 gene. Recently, Chang *et al.* investigated the possibility of reversing or preventing the progression of cardiac manifestation in Friedreich's ataxia. They used a mouse model of FRDA and an adeno-associated viral vector (AAV8) that contained the genetic coding sequence of the FXN gene [164]. Although viral vectors are the most practical gene transfer method, non-viral vectors are also evolving due to their lower toxicity, easier production, and non-immunogenicity [165].

On the other hand, a restriction of using endonucleases to target mitochondria is the risk of targeting other sequences in the mDNA and/or nDNA, which might result in insertions or deletions (INDELs) [157]. The off-target sequences in the nuclear genome, comparable sequences of the binding site for mitoTALENs or mtZFN, can be identified by search in BLASTn. Despite the existence of many mtDNA pseudogenes in the nuclear genome, it is feasible to identify particular mtDNA sequences where the monomers may attach. Amplification resequencing methods can be employed if comparable sequences are detected in the nuclear genome. The goal is to determine whether they have the most significant homology with the mtDNA target site [166].

In the past, mitochondria have only been targeted successfully with three DNA editing platforms: transcription activator-like effector nucleases (TALENs) [167], restriction endonucleases, and zinc finger nucleases (ZFNs) [168]. Despite the structural differences, they are all capable of introducing DSBs at specific sites [169]. In addition, CRISPR/Cas9 (RNA-guided endonucleases) is described as a technique for editing mtDNA in one study [170, 171]. As part of this study, sgRNAs targeting specific loci of the mitochondrial genome were used to edit mtDNA using Cas9 [170]. Since mRNA is mostly imported into mitochondria by tRNA, CRISPR-Cas9-mediated gene editing of the mitochondrial genome faces the great challenge of delivering gRNA through the mitochondrial membrane effectively. Consequently, further analysis of this approach will be required [171]. In the study conducted by Bayona-Bafaluy *et al*., several mitochondrial-targeted restriction endonucleases, including mitoApaLI, were developed. It can differentiate between mice carrying two different mtDNA haplotypes (BALB and NZB) and mtDNA by identifying the presence of an ApaLI site in only one haplotype (BALB) [172]. It provides proof of the principle that mitochondrial endonucleases can be utilized to change mtDNA heteroplasmy by expressing mitoApaLI in cultured hepatocytes. An mtDNA point mutation causing NARP (neurogenic muscle weakness, ataxia, and retinitis pigmentosa disease or Leigh's disease) was targeted with restriction endonuclease SmaI, which led to wild-type mtDNA repopulation [173]. Additionally, using mitochondria-targeted XmaI coding in an adenovirus expression system, the mutant mtDNA was selectively destroyed, which was dependent on both time and dosage. This also led to the restoration of some of the biochemical phenotypes that were caused by the mutation [174].

Research offered evidence that particular restriction endonucleases can be effectively utilized to modify mtDNA heteroplasmy *in vivo*, demonstrating the feasibility of the approach. It is also possible to induce damage to mtDNA, thereby creating models of diseases. For instance, Moraes lab generated mtDNA deletions in aging mice with an inducible form of mitoPstI expressed in various mouse tissues in this regard [175]. As a result, muscle wasting and declines in locomotor activity were observed or mtDNA damage was caused in dopaminergic neurons associated with Parkinson's disease observed in mice [176].

A recent addition to the mtDNA manipulation toolbox is the development of mitoARCUS, based on the *I-CreI* of the LAGLGIDADG meganuclease family [177]. In contrast to *I-CreI*, the newly developed enzyme acts as a monomer and promises potential as a mtDNA editing tool because it can be engineered to be highly specific yet has a comparatively small size (40kDa) and can be delivered by an AAV vector. Results showed that mitoARCUS can be used to manipulate heteroplasmy levels in a cultured cell model as well as mouse models, resulting in the restoration of a wild-type phenotype [177].

Using restriction endonucleases, mitoTALENs, mtZFN nucleases, and mitoARCUS, a brief reduction in mtDNA levels has been observed, but the copy number of mtDNA is eventually recovered [178]. In particular, this happens when the initial levels of mutation are high or a high dose of the gene-editing endonuclease is employed [166]. After recovery, heteroplasmy levels are shifted in favor of the non-targeted (wild-type) variant [159].

It has been suggested to use the polynucleotide phosphorylase (PNPase), which is a native RNA transport enzyme encoded by the *PNPT1* gene and placed in the intermembranous space of mitochondria as a means of delivering nucleic acid into mitochondria [179]. Increasing evidence suggests that PNPase is involved in the import of small RNAs into the mitochondria [180, 181]. In addition, it has been claimed that nuclear RNAse P facilitates the transport of these RNAs into mitochondria in a PNPase-dependent manner by adding the stem-loop sequence to transcripts that normally do not go to mitochondria. However, the delivery of sgRNAs into mitochondria using this strategy has not yet been demonstrated [182]. Using a hybrid guide RNA construct (sgRNA), Hussain *et al.* developed a novel hybrid guide RNA construct (sgRNA) that has been enriched with the 20-nucleotide stem-loop structure of RNAse P at the 50 ends of the sgRNA sequence to address the challenges associated with gene editing tools and gene deletion for mitochondrial diseases. As a result, the population of mtDNA carrying the desired gene region has decreased significantly. As a result, it appears that CRISPR/Cas9 may be able to target mitochondrial DNA *via* PNPase-mediated transport of sgDNA, offering a new way to eliminate disease-causing mutations in mitochondria [183]. To date, mtDNA manipulation has only been limited by the design of nucleases. CRISPR-free mitochondrial base editing may be made possible by the use of RNA-free DddA-derived cytosine base editors. Despite the fact that the ability of this technology to remove mutations in the mitochondrial genome is an exciting prospect, this preclinical technique has not yet been tested in even the most basic animal models [21, 184].

A serious concern in all of these approaches is the safe delivery of mitochondrial nucleases if we can treat mitochondrial disease by transiently exposing patients to a therapeutic gene product that does not trigger the activation of oncogenes [185].

A branch of gene therapy called in-utero gene therapy (IUGT), which is still in its infancy, aims to prevent the early pathogenic alterations that occur in inherited genetic diseases. IUGT's benefits, such as addressing irreversible pathogenic alterations, may outweigh the drawbacks of postnatal gene therapy, such as access to a high number of proliferative stem/progenitor cells in many organs, small fetal size, and reducing disease complexity before birth [186].

Furthermore, embryo manipulation techniques like MRT have evolved in recent years with the ability to eliminate mutated mtDNA [187]. It is important to perform preimplantation genetic tests and select embryos with low mtDNA mutation loads, which are less likely to develop clinical symptoms in the future [188]. The MRT process involves removing chromosomes from an enucleated oocyte from a healthy donor and replacing them with the chromosomes of the would-be mother at risk of transmitting mutated mitochondrial DNA, resulting in the donor egg contributing only its mitochondrial DNA to the embryo [189].

MRT, also called mitochondrial donation, is a newly developed technique to prevent transmission of mtDNA. In fact, MRT decreases the risk of transmission efficiently; however, it does not eliminate the risk [190]. In this method, the nuclear DNA of the affected woman is transferred to a donated oocyte or zygote *via* two partially different techniques, namely pronuclear transfer (PNT) or maternal spindle transfer (MST) [191]. The principle of PNT is transferring the pronuclei of an *in vitro* fertilized zygote originating from intending parents to a donor zygote containing healthy mtDNA and lacking pronuclei [192]. A donor zygote is created *via in vitro* fertilization of a donor oocyte by the intending father’s sperm. In contrast to PNT, the MST procedure initiates before fertilization, and the metaphase II spindles of the donor oocyte are replaced with the intending mother’s metaphase II spindles. Then, the newly created oocyte is fertilized by the intending father’s sperm [192].

A recent study in the USA on genetic counselors showed that the majority of the participants agreed with the MRT clinical trial because positive results of clinical trials can lead to insurance coverage of mitochondrial donation. Thus, a clinical investigation can contribute to making MRT financially practical [193]. However, only half of the genetic counselors supported the approval of MRT clinical use [193]. MRT seems to be a promising approach, even though there are restrictions such as ethical issues and a risk of carryover mtDNA, which may increase due to selective replication and genetic drifts. Hence, in recent years, Fan *et al.* studied this field and suggested two ways to induce mitophagy to eliminate mtDNA carryover and decrease the following heteroplasmy [194, 195]. First, they suggested using mRNA for labeling the outer membrane of donor zygote mitochondria [195]. The second method was taking advantage of signaling events involved in mitophagy. In fact, they suggested direct inducement of forced mitophagy by expression of SQSTM1 and its MAP1LC3B-binding domain (Binding) on the outer membrane of mitochondria [194].

In conclusion, accurate genetic counseling is essential for all patients with mitochondrial disease, and the risks and advantages of all feasible options should be discussed to allow patients to make the most appropriate decision [109]. The permission of rigorous clinical research can minimize the MRT critical risks and expand the range of available options.

### 3.6 Mitochondrial Diseases Transmission Prevention

Mitochondrial disorders can originate from defects in the nuclear genome or mtDNA. Therefore, there are different approaches to prevent the transmission of these disorders [109]. For example, the options for disorders with nuclear genetic defects are the same as other nuclear genetic disorders [109], including counseling, preimplantation genetic diagnosis (PGD), and prenatal testing [192]. However, for disorders with maternal inheritance of mtDNA, the options and challenges are different [192]; for instance, heteroplasmy and genetic bottleneck. A cell might obtain homoplastic (only wild-type or mutant) or heteroplastic (a combination of wild-type and mutant) mtDNA. The proportion of mutant mtDNA and its proximity to a threshold to determine its phenotype, as mitochondrial dysfunction, only manifests when the proportion of mutant mtDNA exceeds a critical threshold, typically >80%, in an affected cell [4]. Hence, the options depend on mutation level as well as the type of mutation harbored for women with heteroplasmic mtDNA diseases [109]. Genetic diagnosis and counseling are both significantly important for this group. After counseling, women might decide to remain voluntarily childless, adopt a child, or use oocyte donation [192]. In addition, preimplantation genetic diagnosis (PGD) and prenatal testing are other options developed in recent years [192]. PGD can be a suitable option for some cases with heteroplastic mtDNA mutations, although some cases cannot take advantage of it [109]. In this procedure, an embryo with the lowest level of mtDNA mutation is identified and selected for transfer into the uterus [196]. Only in a restricted number of embryos, the mutation level is low enough that it is impractical to cause a severe mitochondrial disorder [197].

## 4 LIMITATIONS

Some characteristics of mitochondrial diseases hamper some of these disorders, taking advantages of developing diagnostic and therapeutic methods, including genomic analysis and gene therapy. In the field of diagnosis, several natural characteristics of human mtDNA make the diagnosis of mitochondrial disorders more complex. Tissue heteroplasm, which is the co-existence of wild type and mutated mitochondrial DNA in an individual, might raise the need for reads with an adequate depth to detect low-level heteroplasmy. Since the mtDNA does not have the advanced DNA repair mechanisms that are present in the nuclear DNA (nDNA), rare but benign variations in mtDNA might be encountered relatively frequently in individuals (increased mutability). Therefore, additional factors, including rare haplogroup signatures, conservation and (predicted) consequence along with rarity, must be considered when filtering out the variants [39]. The nuclear mitochondrial DNA segments (NUMT) can be very similar in sequence to mtDNA. This can create problems when trying to map and analyze mtDNA and can impact the accuracy of measuring heteroplasmy. The possibility of affecting the process of mapping at the start/end site of mtDNA is due to the circular structure of mtDNA. Additionally, in the case of changing the ratio of some heteroplasmic variations over time (temporal heteroplasmy variations existence), sampling from multiple tissues is required [39].

Since in several PMDs, the central nervous system is involved, which is difficult to target safely and efficiently, performing gene therapy encounters many challenges. Up till now, no *in vivo* intravenous genetherapy product intended to target the brain has been approved by FDA despite numerous clinical trials exploring this approach [198]. The issue with intravenous routes of administration is their requirement for high doses of AAV vector, which was demonstrated to cause toxicity. Likewise, administration *via* intracranial routes, which is more efficient than IV, has raised other challenges, including being invasive and the risk of injuring the medulla [198].

## CONCLUSION

In the last few years, significant advances have been made in understanding mitochondrial disease and improving our ability to diagnose, treat, and prevent it. For some patient groups, there are currently targeted treatments. Furthermore, for families with mitochondrial disease, *in vitro* fertilization offers new reproductive options. Despite this, our research needs to focus on the future. Understanding mitochondrial disease mechanisms is essential for understanding why specific patients exhibit tissue-specific manifestations. Thus, by combining the results of early WGS, clinical evaluation, and serum biomarker stratification, it is possible to create a less invasive diagnostic process for patients, increase diagnostic yield, provide individual prognosis and collective comprehension of mitochondrial biology, and eventually pave the way for significant therapeutic advancements.

As we move forward, the most important challenge in NGS technology is accurately determining which variants are pathogenic from other benign variants between individuals and where they are located in the genome. The key to interpreting these variants and expediting the diagnosis of mitochondrial diseases is the development of standardized guidance for the integration of clinical, functional, and “multi-omics” data and variant interpretation.

The use of multi-omics is becoming more prevalent and is replacing single gene product studies such as qPCR and RT-PCR. There is a great deal of information that can be gained from these assays with regard to the pathomechanism of the causative variant, which can help establish an unexpected diagnosis. As a result of the increased diagnostic value of transcriptomic, proteomic, and metabolomic analyses, the systematic collection of patient biological samples is encouraged, but also, crucially, a better understanding of mitochondrial biology and mitochondrial disease pathology is gained. In addition, to advance research, clinical and basic scientists, the pharmaceutical industry, and patient organizations must work closely together.

Recent developments have opened possible routes for novel therapies for the treatment of mitochondrial disorders. Studies on AAV-mediated gene therapy to treat mitochondrial disorders caused by mutations in nuclear genes, fixing the disorders at their very roots, are underway. Mitochondrial genome editing approaches have shown promising results in cell and animal models, and mitochondrial augmentation or transplantation techniques have yielded intriguing results in a few patients. However, it is likely that it will still take several years until the usefulness, safety, and efficacy of these approaches have been demonstrated and they will become the standard repertoire for the treatment of mitochondrial diseases. Nevertheless, while the prospects for patients suffering from disorders related to mitochondrial dysfunction were bleak not even ten or fifteen years ago, there is now some justification for the hope that mitochondrial disorders will become much more manageable with treatments that, at the very least, will yield a marked improvement in quality of life of affected individuals.

## AUTHORS’ CONTRIBUTIONS

It is hereby acknowledged that all authors have accepted responsibility for the manuscript's content and consented to its submission. They have meticulously reviewed all results and unanimously approved the final version of the manuscript.

## Figures and Tables

**Table 1:** Some of the current clinical trials in mitochondrial disorders initiated in 2023.

**Interventions**	**Mechanism**	**Status**	**Conditions**	**Phase**	**Location**	**Purpose of Study**	**Subjects**	**Trial Number**
**Coenzyme Q10**	Antioxidant	Not yet recruiting	Prader-Willi Syndrome	Phase 2	Canada	To clarify the influence of Coenzyme Q10 on cellular metabolism in individuals with PWS.	N= 14 Age= child^I^, adult^II^ Sex= female and male	NCT03831425
**Omaveloxolone (SKYCLARYS)**	Enhances mitochondrial biogenesis	Not yet recruiting	Friedreich Ataxia	Phase 1	United States	To assess the tolerability, safety, and pharmacokinetics of omaveloxolone in pediatric patients suffering from Friedreich's Ataxia	N=20 Age= child Sex= female and male	NCT06054893
**N-Acetylcysteine**	Antioxidant/restoring nitric oxide production	Recruiting	Mitochondrial Disease	Phase 1	United States	To identify the safest and most effective dose of N-Acetylcysteine.	N=18 Age= adult, older adult^III^ Sex= female and male	NCT05241262
**Nicotinamide**	Enhances mitochondrial biogenesis	Not yet recruiting	Friedreich Ataxia	Phase 2	Austria/ France/ Germany/ Italy/ Spain/ United Kingdom	To present clinical evidence supporting the effectiveness and safety of nicotinamide in individuals with Friedreich ataxia.	N= 225 Age= adult, older adult Sex= female and male	NCT03761511
**Nicotinamide Riboside (NR)**	Precursor of NAD+/increases mitochondrial number and mitochondrial function	Recruiting	Mitochondrial Myopathy	Phase 2	United States	To investigate the impact of Nicotinamide Riboside on adult-onset symptoms of mitochondrial myopathy	N=34 Age= adult, older adult Sex= female and male	NCT05590468
**OMT-28**	Enhance mitochondrial function	Recruiting	Primary Mitochondrial Disease	Phase 2	Germany/ Italy	To study the effects of OMT-28 on patients with primary mitochondrial disease	N=32 Age= adult Sex= female and male	NCT05972954
**NR082 Injection**	Gene Therapy	Recruiting	Leber Hereditary Optic Neuropathy	Phase 1/ Phase 2	United States	To assess the effectiveness and safety of NR082 in treating LHON associated with ND4 mutations	N=18 Age= adult, older adult Sex= female and male	NCT05293626
**NFS-02 Injection**	Gene Therapy	Recruiting	Leber Hereditary Optic Neuropathy	Phase 1/ Phase 2	United States/ China	To assess how safe and well-tolerated NFS-02 is and to look at its early effectiveness in treating LHON that is caused by a mutation in the mitochondrial ND1 gene	N=18 Adult, Age= older adult Sex= female and male	NCT05820152
**MNV-201**	Cell therapy produced by MAT	Recruiting	Mitochondrial Diseases/Pearson Syndrome	Phase 1	Israel	To assess the safety and therapeutic benefits of transplanting MNV-201 in children suffering from Pearson Syndrome	N= 5 Age= child, adult Sex= female and male	NCT06017869
**Autologous USC (urine-derived stem cell) mitochondrial oocyte transplantation**	MAT and mitochondrial transplantation	Enrolling by invitation	Female Infertility / Mitochondrial disorder	-	China	To investigate the effectiveness and safety of autologous mitochondrial transplantation of stem cells derived from urine in elderly women with a poor prognosis	N= 40 Age= adult, older adult Sex= female only	NCT06020742

**Note:**^I^Child= birth – 17 years old.

^II^Adult= 18 – 64 years old.

^III^Older adult= above 65 years old.

## LIST OF ABBREVIATIONS

AAV: Adeno-associated Viruses
ATP: Adenosine Triphosphate
CNVs: Copy-number Variants
DBPC: Double-blinded, Placebo-controlled
FGF21: Fibroblast Growth Factor 21
GC/MS: Gas Chromatography-mass Spectrometry
HSPCs: Hematopoietic Stem and Progenitor Cells
IEM: Inborn Errors of Metabolism
IUGT: In-utero Gene Therapy
LC-QTOF: Liquid Chromatography Quantitative time-of-flight
LHON: Leber Hereditary Optic Neuropathy
MAE: Monoallelic Expression
MAT: Mitochondrial Augmentation Technology
MDVs: Mitochondrial-derived Vesicles
mitoRE: mitochondria-targeted Restriction Endonuclease
MRTs: Mitochondrial Replacement Techniques
MS: Mass Spectrometry
MST: Maternal Spindle Transfer
mtDNA: mitochondrial DNA
NAC: N-acetylcysteine
nDNA: nuclear DNA
NGS: Next-generation Sequencing
OMIM: Online Mendelian Inheritance in Man
ONT: Oxford Nanopore Technologies
OXPHOS: Oxidative Phosphorylation
PacBio: Pacific Biosciences
PGD: Preimplantation Genetic Diagnosis
PMD: Primary Mitochondrial Disease
PNT: Pronuclear Transfer
PPAR: Peroxisome Proliferator-activated Receptor
rGS: rapid Genome Sequencing
RNA-seq: RNA Sequencing
ROS: Reactive Oxygen Species
SCEH: Short-chain enoyl-CoA hydratase
TALENs: Transcription Activator-like effector Nucleases
VLCADD: Very Long-chain acyl-coenzyme A Dehydrogenase Deficiency
VUSs: Variants of Uncertain Significance
WES: Whole Exome Sequencing
WGS: Whole Genome Sequencing
ZFNs: Zinc Finger Nucleases
